# Evaluation and Comparison of Stress in Divergent and Convergent Collar Designs of Implants With Different Bone Densities: A Finite Element Study

**DOI:** 10.7759/cureus.36550

**Published:** 2023-03-22

**Authors:** Gracy Panmei, Arunoday Kumar, Suchetana Basak, Seltun S Anal, Rajesh Nongthombam, Braj B Mall

**Affiliations:** 1 Prosthodontics and Crown & Bridge, Private Practice, Imphal, IND; 2 Prosthodontics and Crown & Bridge, Dental College, Regional Institute of Medical Sciences (RIMS), Imphal, IND; 3 Periodontics, Dental College, Jawaharlal Nehru Institute of Medical Sciences (JNIMS), Imphal, IND; 4 Oral and Maxillofacial Surgery, Dental College, Regional Institute of Medical Sciences (RIMS), Imphal, IND

**Keywords:** finite element method (fem), bone densities, convergent collar design (ccd), divergent collar design (dcd), finite element analysis (fea)

## Abstract

Background: The failure and the success rate of an implant depends on biomechanical factors, esthetics and painless sterile implant surgery conditions, out of which stresses applied to the bone and its surrounding, bone-implant interface, material characteristics of the implant used and the strength of the bone and its surrounding are the important factors. This study aimed to evaluate the stress distribution of divergent collar design (DCD) and convergent collar design (CCD) implants placing them in four different densities of the bone (D1, D2, D3 and D4). The evaluation of the stress distribution of DCD and CCD was performed using the 3D finite element method (FEM), by placing them in four different bone densities. In addition to this, a comparison of the effect of DCD and CCD in terms of stress distribution in the bone was also done.

Materials and methods: The software used to process the geometric characteristics of the missing first molar in the mandibular section were Ansys, version 19.2, CATIA, version 5, and Solidworks (Dassault Systèmes). Using these software, three models were designed and successfully restored using an all-ceramic crown implant. The first model was a geometric model of the first molar mandibular bone section, the second model was a cylindrical implant (4x10 mm) with a DCD and CCD, and the third model had titanium alloy (Ti-6Al-4V) properties incorporated into the implant.

Results: The D1 bone model showed the lowest stress concentration compared to D2, D3, D4. The DCD showed the lower stress and strain concentrations as compared to the CCD in the contiguous crestal bone in all the densities of the bone in both vertical and lateral or oblique loadings. The DCD with the D1 bone showed the least stress concentration around the crestal bone region. The results of this study also showed that the maximum von Mises stress was observed in the crestal region or the neck of the implant for both the convergent and divergent collar implant designs in all the four densities of the bone.

Conclusion: Before a patient trial of a new implant design or a new implant material, finite element analysis (FEA) gives us a clear picture of what will be the patient bone response when an implant will be placed and loaded. FEA also gives us an opportunity to test a new implant material without putting a patient at risk. In this study, four different types of bone were incorporated with two different implant collar designs. Each implant assembly was subjected to vertical as well as oblique forces. The response of each bone type for the titanium alloy implant was recorded. A color-coded response for the magnitude and the location of the maximum stress received by the bone was observed. Maximum stresses were seen in the crestal region. As this is a computer-based model, dynamic loading was not possible. This study provided us with the possible outcome in patients under a static load. Further studies can be conducted in vivo to record dynamic loading responses as well as long-time loading responses.

## Introduction

Dentistry in the modern era has advanced to a great extent. Replacement of missing teeth with the osseointegrated supported prostheses called dental implants has been introduced. The aim of implant dentistry is to rehabilitate a patient’s missing teeth with dental implants in order to achieve normal mastication, esthetics, phonetics, health and comfort [1]. But all the dental implant prostheses are not successful. A recipient’s quality and quantity of bone, techniques of surgery while placing the implant, designs and surface of the implant fixture, recipient-related local factors, etc., are the determining factors for the good prognosis of dental implants. Therefore, the longevity of dental implants for the rehabilitation of missing teeth carries many challenges in clinical situations [2]. In the literature, a prognosis of about 95% is found for successfully placed mandibular dental implants and 90% for maxillary implants. Despite the best materials used for implants, implant fixture designs, and techniques of surgery, implant failures do occur occasionally, and hence, strategies to prevent the failure of an implant should be planned [3].

In the treatment planning for a dental implant, quality and quantity of the recipient’s bone is utmost important to be considered for a better prognosis. A thick, dense cortical bone is found in the mandibular anterior alveolar arch, followed by mandibular posterior and anterior maxillary alveolar arches. The posterior maxillary alveolar bone has the lowest density [1].

The four standard bone densities (D1, D2, D3, D4) to place the implants were given by Lekholm and Zarb in 1985. As reported by Jaffin and Berman, the D4 bone showed 55% of failure rate [4]. The reaction of the bone around implants is very important to know the prognosis. The mechanical forces applied to dental implants while biting or chewing and their directions and pattern in the alveolar bone are very important to understand the biological tissues reactions. For a successful implant surgical technique, factors like initial retention and stability, osseointegration, protection from stress, prevention of microbial growth at the surgical or implant site, biological width, etc., have to be considered. In addition to this, the design of the collar also has to be taken care of as a good design can prevent the infiltration of microbes and hence prevent it from failing [5].

The stress can be originated at the implant crestal module, where the crestal bone makes contact with the implant. The initial bone quality and quantity are the reason for the mechanical immobilization in the healing process and also give a better stress distribution and transmission at the implant-body interface [6]. The crest module region has a high concentration of mechanical stress, where resorption of the bone usually happens. This happens due to excess loading, non-stimulation of the bone, or infiltration and growth of microbes at the implant site [7]. The design of the implant crest, like divergent collar design (DCD) and convergent collar design (CCD), may improve the bone-implant integrity in terms of stability and osseointegration by reducing the excess stress on the bone and initiates bone stimulation [8].

For designing the best dental implants, stress transferred to the surrounding bone has to be precisely analyzed [9]. In implantology, a simulation method called finite element analysis (FEA) is used to study the stress pattern by applying numerical techniques called finite element method (FEM) [10]. It is also able to locate the origin, magnitude and direction of the relevant forces acting on a surface. In addition to this, it can also assign and measure the stress points.

In the bone around an implant, different mechanical stresses such as von Mises stress that comprises tensile stress, compressive stress and the maximum shear stress can be found [11,12]. von Mises stress measurement gives the yielding point of various materials. The tensile stress is the maximum principal stress, with the minimum being the compressive stress. The use of these stresses is considered good for studies of the implant and the bone that has both ductile and brittle properties [13]. Hence, to design a healthy crest module design, considering the influencing factors like critical stress and bone stimulation, we evaluated effects of two different implant collar designs (convergent and divergent) on stress distribution in D1, D2, D3 and D4 quality of bones with three-dimensional FEA, wherein D1 is a dense cortical bone found in the anterior mandible, D2 is a porous cortical and coarse trabecular bone found in the anterior and posterior mandible, D3 is a porous, cortical fine trabecular bone found in the anterior and posterior maxilla, and D4 is a fine trabecular bone found in the posterior maxilla.

In the present study, the pattern of load distribution like vertical, horizontal or oblique loading in a section of the mandibular bone with a missing first molar was used, which was restored with a ceramic crown-based implant. Also, a geometrical model of the first mandibular molar area bone section, a cylindrical implant (4x10 mm) with a divergent and convergent collar design and an implant model with titanium alloy (Ti-6Al-4V) properties incorporated were developed in this work.

## Materials and methods

A 3D bony section of a mandibular model was prepared and a dental implant was designed with software CATIA, version 5 (Dassault Systèmes, Vélizy-Villacoublay, France) and SolidWorks (SolidWorks Corp., Waltham, MA). The models were subjected to pass through finite element stress analysis with software Ansys, version 19.2 (Ansys, Inc., Canonsburg, PA) and SolidWorks; this determined the response of the bone density to a particular collar design.

**Figure 1:**
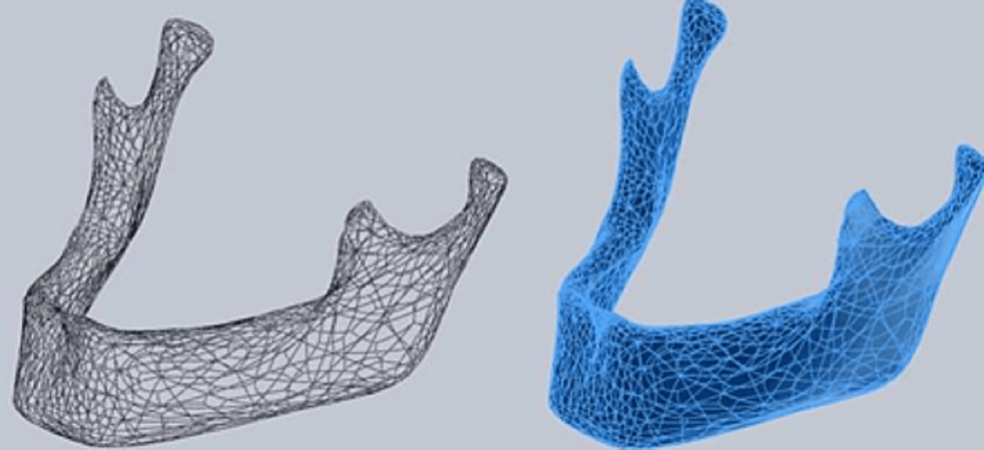
Framework of the human mandible

The first step to construct the geometry of the model involved modelling different types of bone densities (DI, D2, D3, D4), with similar morphological architect of the bone as in the mandibular first molar region area using CATIA V5 software. Figure 1 shows the framework of the human mandible.

**Figure 2:**
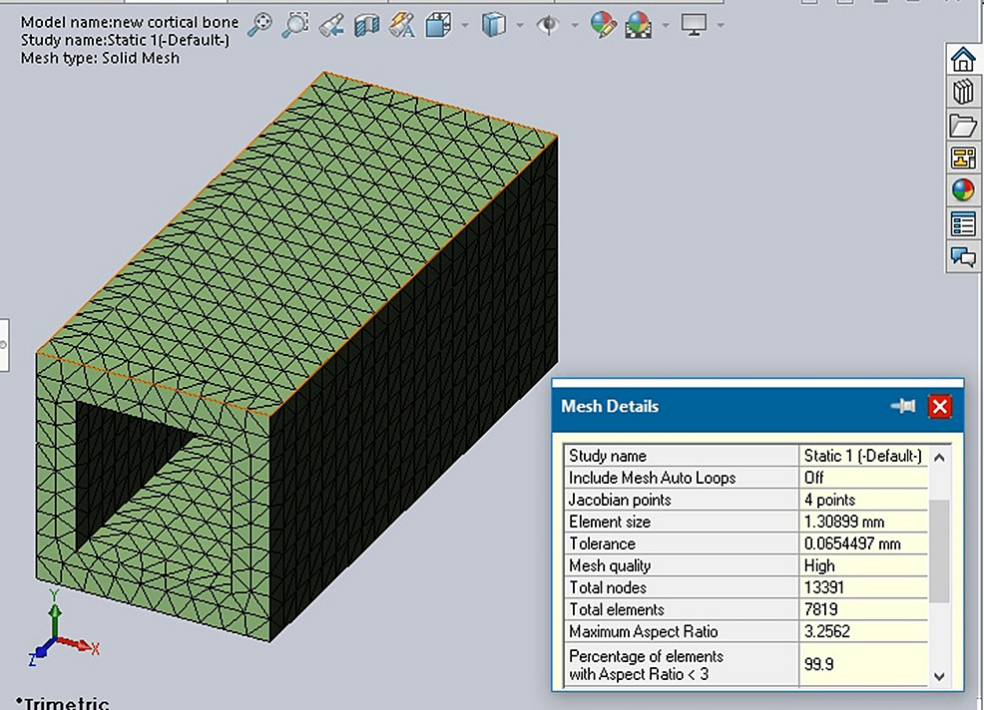
Cortical bone mesh

**Figure 3:**
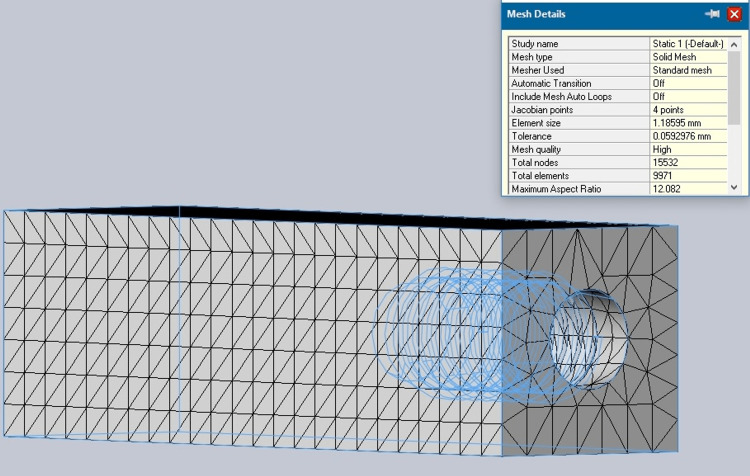
Cancellous bone mesh

Cortical and cancellous bone properties were incorporated into the bone model as shown in Figures 2, 3.

**Figure 4:**
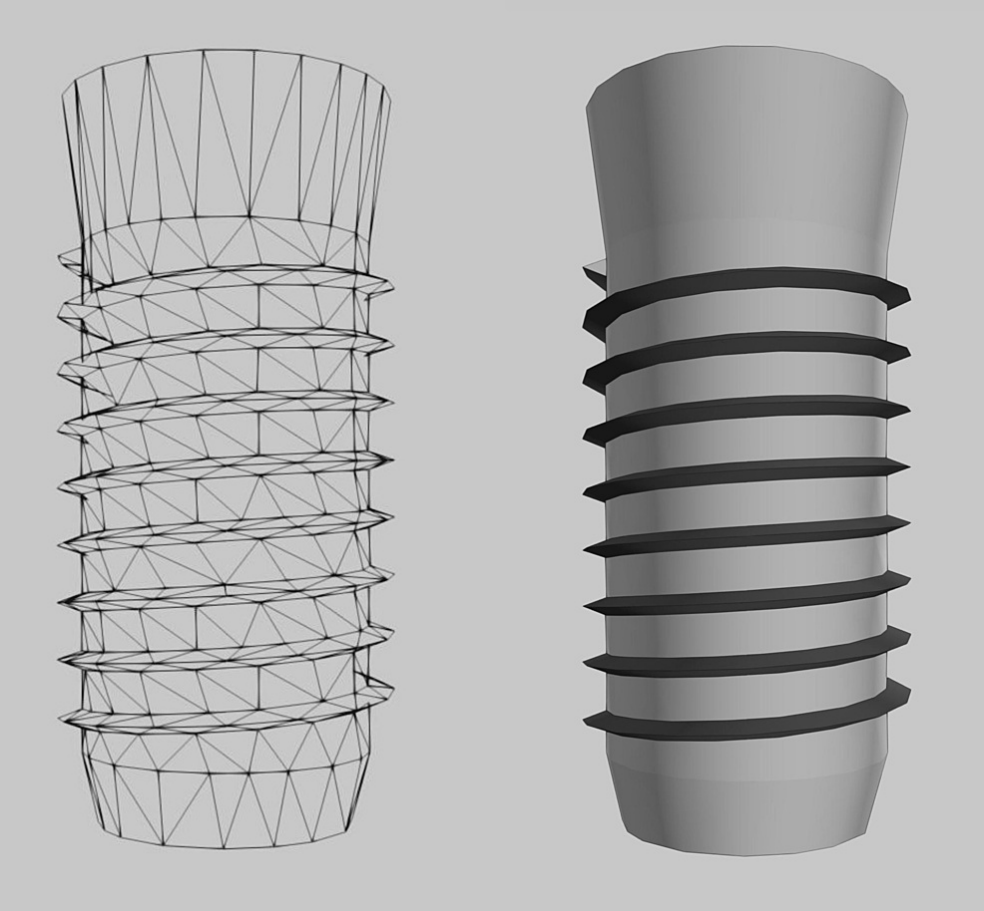
Wireframe for the dental implant model

The average height and width of the mandibular section model were 28 and 12mm. Figure 4 shows the wireframe for the dental implant model, which was designed so as to have a fixture length of 10mm, and body and apical diameter of 4 and 3.5mm, respectively.

A V-shape thread was incorporated into the implant model that encircled the implant body with a depth of 0.36mm, pitch 0.8mm and width 0.25mm [3].

**Figure 5:**
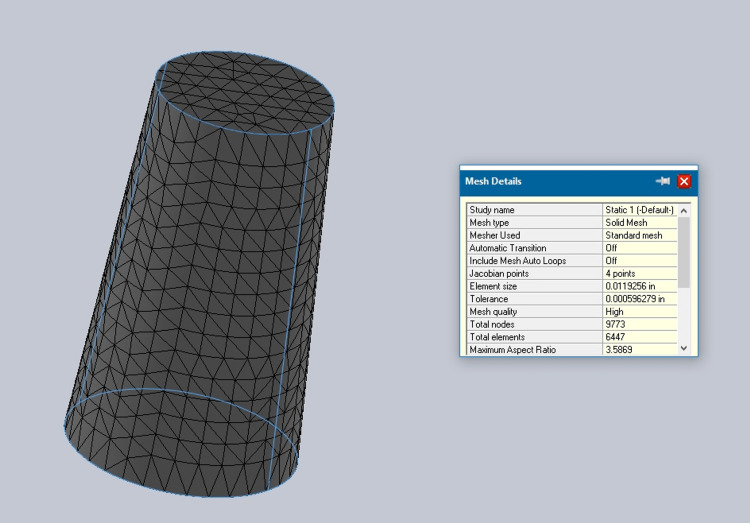
Abutment mesh

**Figure 6:**
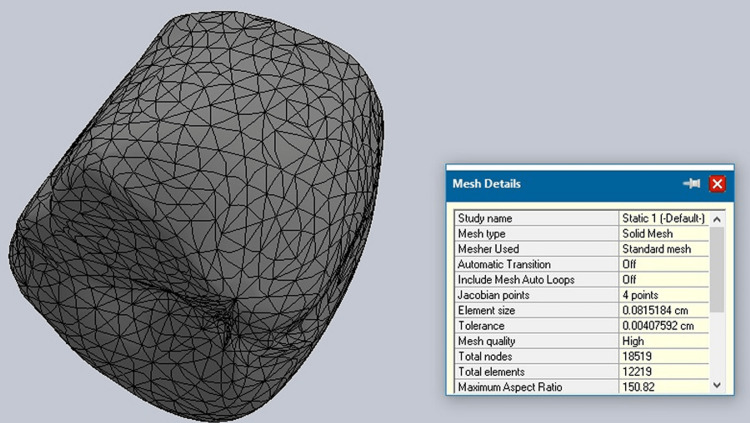
Molar mesh

The two implant collar designs (DCD and CCD) were also designed with SolidWorks and had the same height (2mm) and same diameter (4mm) at the implant collar interface. The diameter of the implant in the collar abutment interface model for CCD was 3.5mm and for DCD was 4.5mm. An abutment conical and convergent in shape, and having a diameter of 3mm at the junction of the abutment and collar with a height of 5mm was designed along with a porcelain prosthetic crown for the missing mandibular first molar replacement. All units of the models were assembled and subjected to abutment mesh and molar mesh with the SolidWorks software as shown in Figures 5, 6.

**Figure 7:**
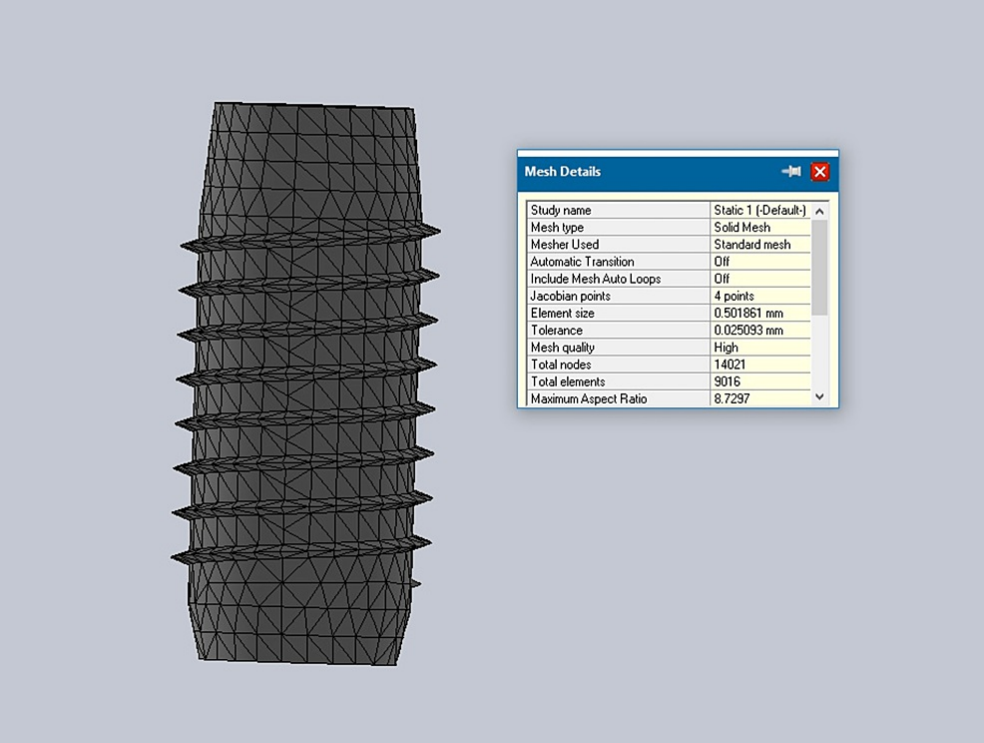
Convergent collar implant mesh

The convergent collar implant mesh is shown in Figure 7, which shows 9016 elements and 14,021 nodes.

**Figure 8:**
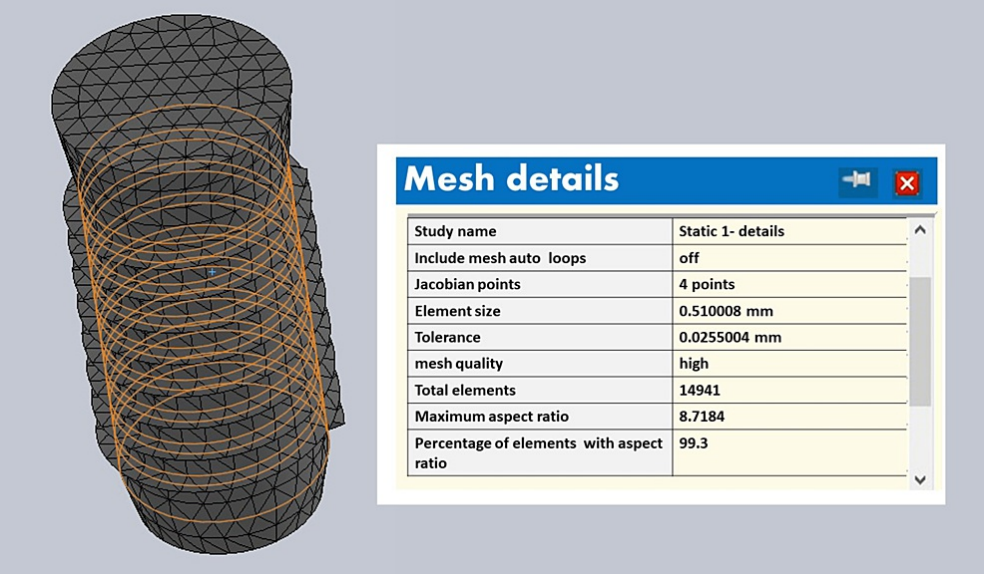
Divergent collar implant mesh

The divergent collar implant mesh is shown in the Figure 8, which shows 9639 elements and 14,941 nodes.

**Figure 9:**
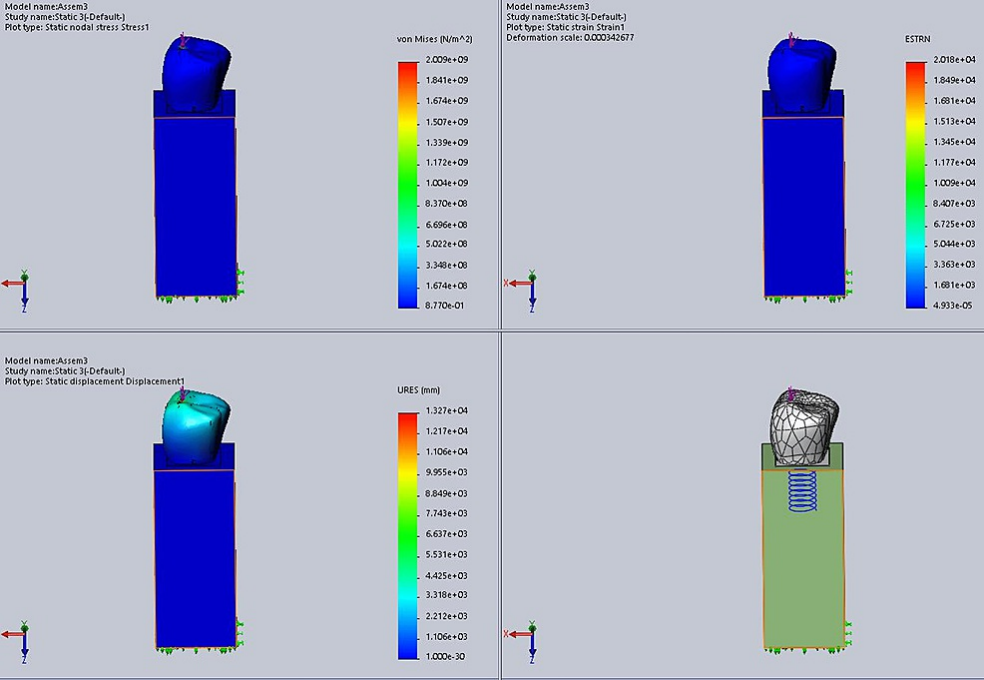
A schematic model representing the application of load in the assembly

After designing the geometrical models, they were fixed at the base and supported at the distal and inferior side of the mandibular bony section to mimic the action of the muscles and ligaments. Then, a vertical force of 400N was applied at the centric fossa and a lateral force of 400N was applied onto the lingual inclines of the buccal cusp of the molar teeth in the linguo-buccal direction at a 30° angle. Basically, two implant models were prepared for each density (DI, D2, D3, D4), with the divergent and convergent implant collar designs, which were tested for the von Mises stress by applying a vertical and lateral or oblique load of 400N on each model. The application of the load is shown by a schematic diagram in Figure 9. The von Mises stress was analyzed with the Ansys software.

The von Mises stress value measures the point of deformation in dental implants ductile in nature. The failure of the dental implant is seen when the von Mises stress is greater than its yield strength [14]. Therefore, it is very important to analyze and evaluate the origin and distribution of stress in the implant material.

The analysis of models was done using the finite element method; the properties of materials used in this method are summarized in Table 1.

**Table 1:** Material properties used in the finite element method Reference [9]

Material	Modulus of elasticity (GPa)	Poisson’s ratio
Cortical bone	14.8	0.35
Cancellous bone		
D1	9.5	0.3
D2	5.5	0.3
D3	1.6	0.3
D4	0.69	0.3
Titanium (implant, abutment)	110	0.35
Porcelain	82.8	0.35
Mucosa	10	0.40

Inclusion/exclusion criteria

Models whose properties matched the material properties enlisted in Table 1 were included in the study, as these values are the average values seen in the normal living bone, alveolar mucosa, titanium implants and porcelain teeth. Models whose properties did not match were excluded.

## Results

**Figure 10:**
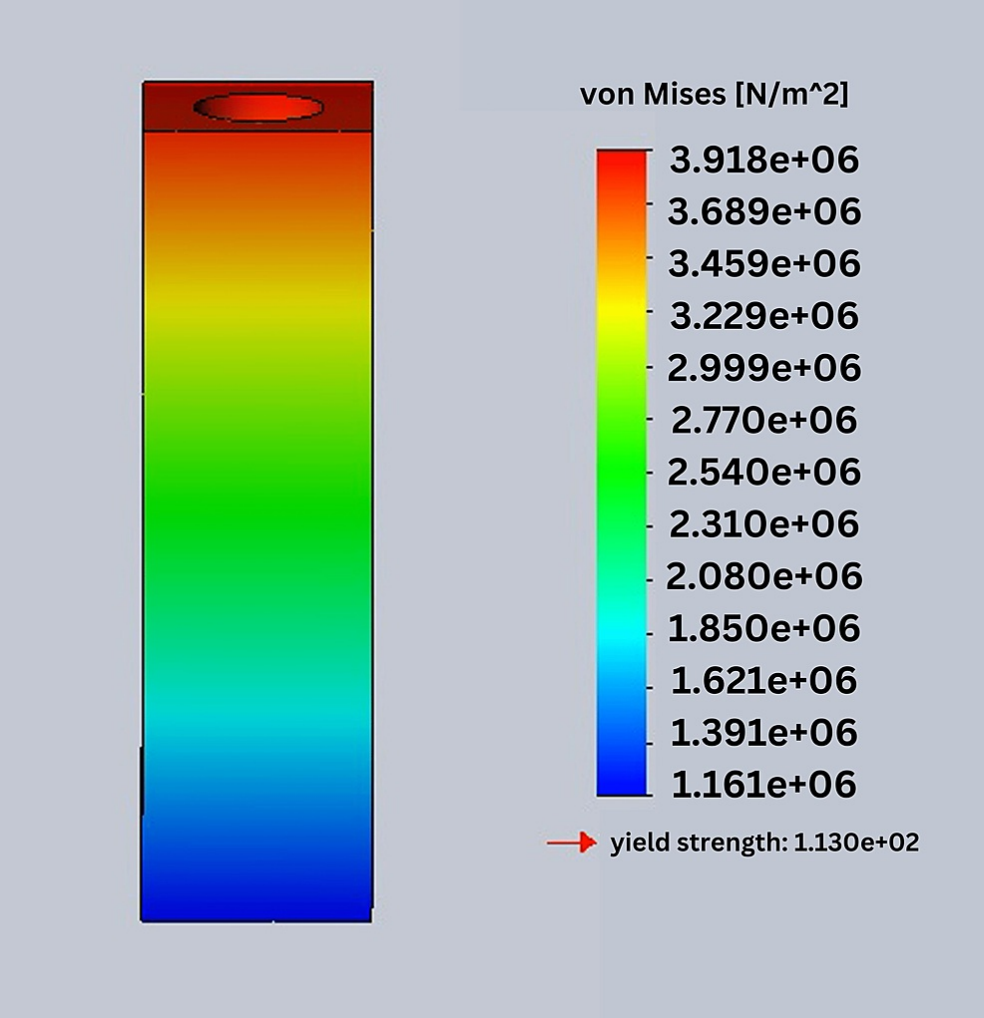
von Mises stress field for the D1 divergent collar with vertical loading

**Figure 11:**
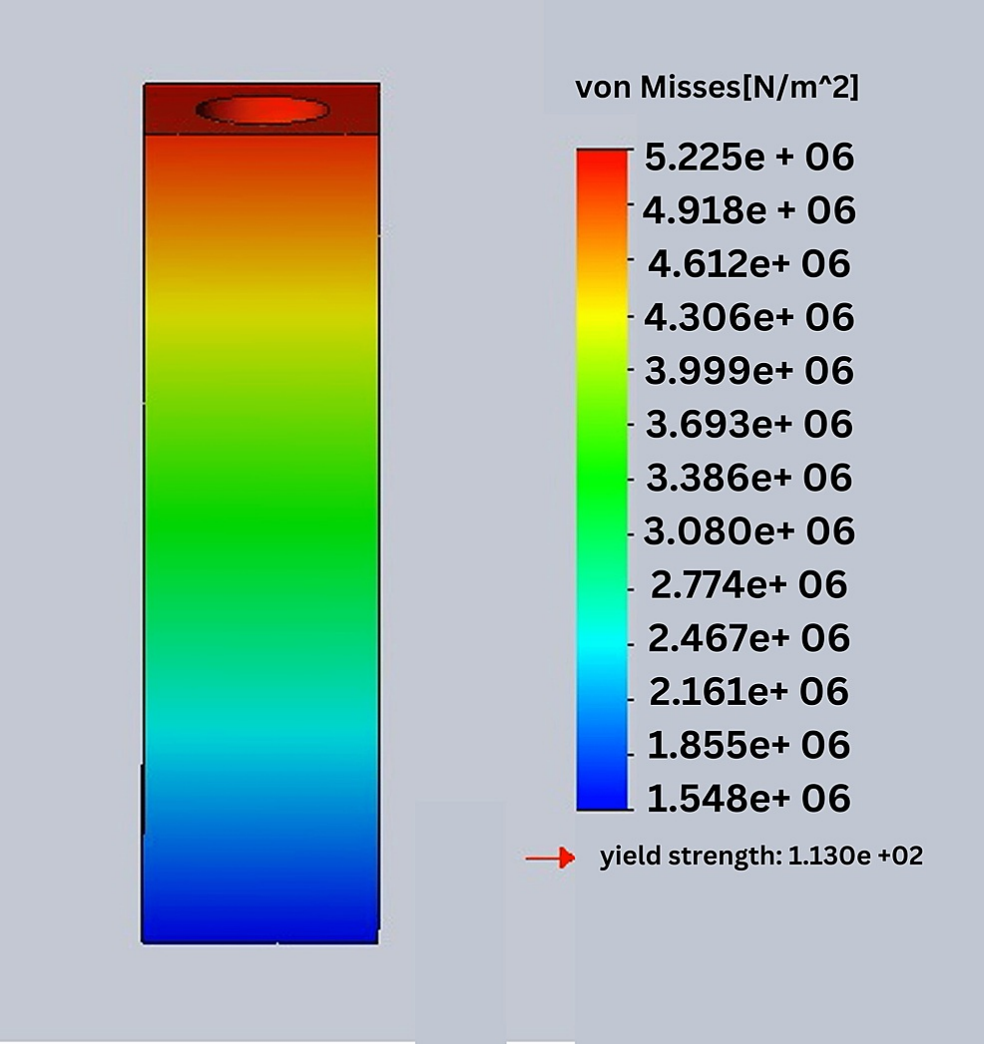
von Mises stress field for the D1 convergent collar with vertical loading

**Figure 12:**
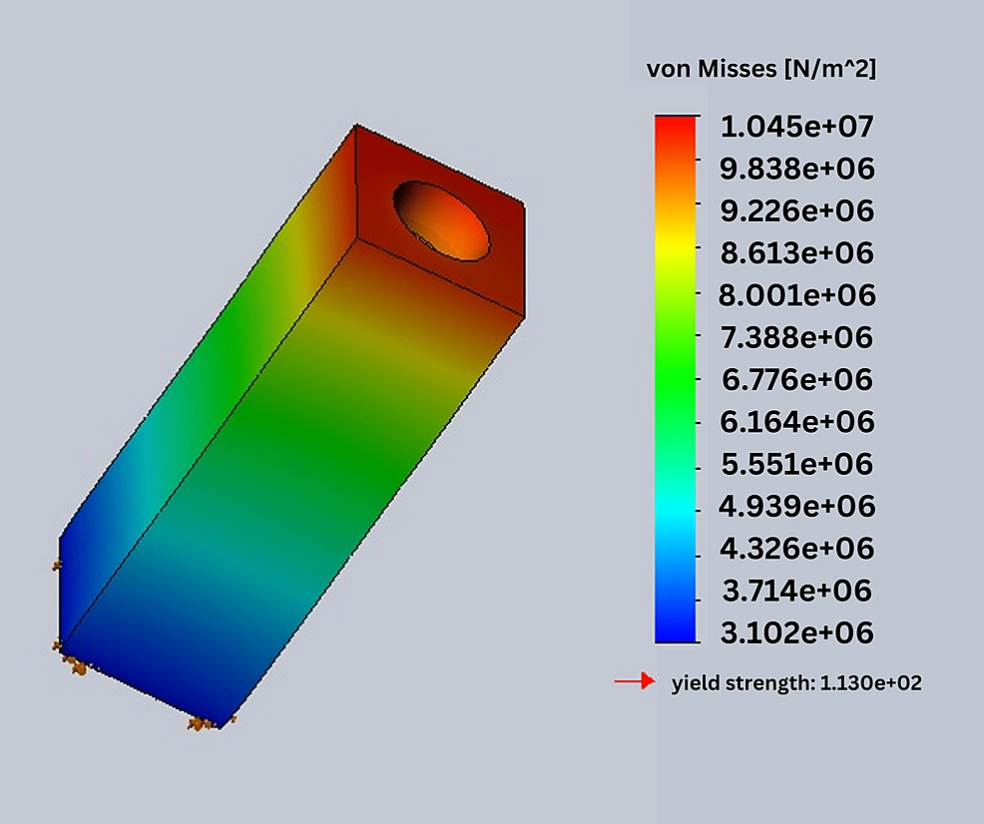
von Mises stress field for the D1 convergent collar with lateral or oblique loading

**Figure 13:**
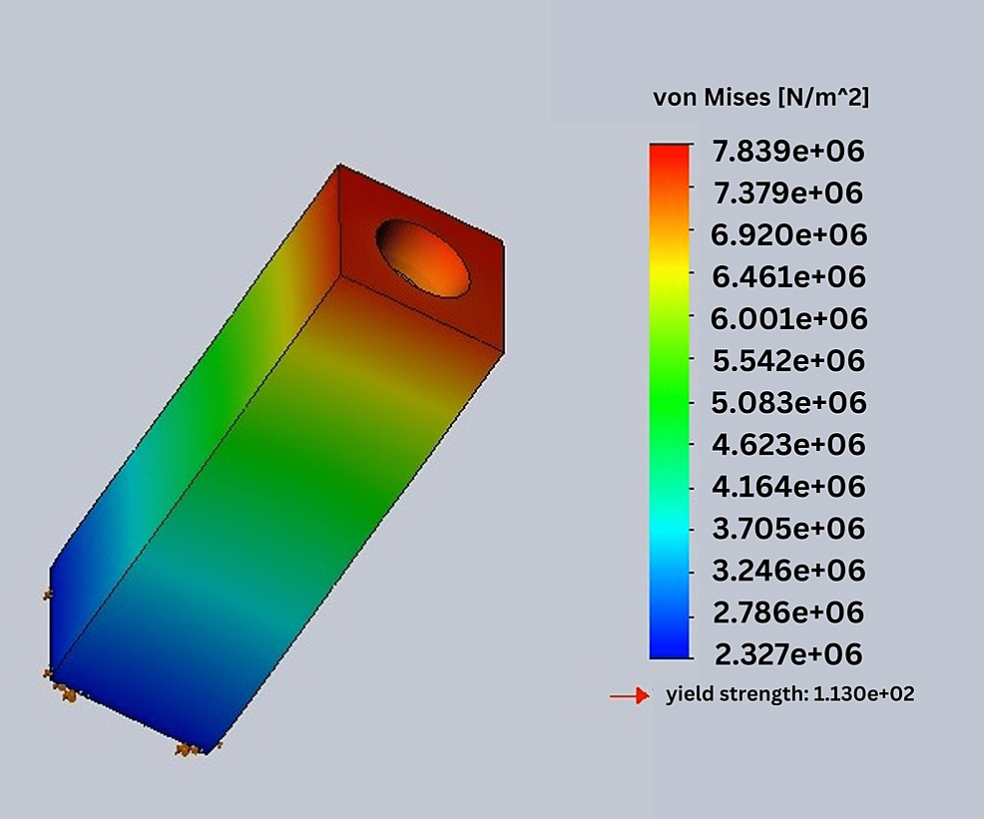
von Mises stress field for the D1 divergent collar with lateral or oblique loading

In this study, stress was analyzed using Ansys software. In this process, von Mises stress field traces could be observed in the form of color-coded bands as shown in Figures 10-13. The range of stress value is marked by different color bands, given in Newton per meter square (N/m^2^). The minimum and maximum stress is marked by blue and red color bands, respectively. It was observed that all the models had the maximum stress at the crest of the bone and collar of the implant. The von Mises stress field for the D1 divergent collar with vertical loading is shown in Figure 10 and the D1 convergent collar with vertical loading is shown in Figure 11. During oblique loading, the D4 bone with the convergent collar showed the maximum stress concentration while the D1 divergent showed the minimum stress concentration. During oblique loading, the D4 bone with the convergent collar showed the maximum stress concentration while the D1 divergent showed the minimum stress concentration. Overall, the D1 bone with the divergent collar showed the minimum stress concentration and the D4 bone with the convergent collar showed the maximum stress concentration in the crestal bone region during the vertical and oblique loading. The stress concentration for vertical loading was lesser than that for the oblique loading. The von Mises stress field for the D1 convergent collar with lateral or oblique loading is shown in Figure 12 and for the D1 divergent collar with lateral or oblique loading is shown in Figure 13.

The D1 bone shows the minimum stress concentration compared to other bone densities. The divergent collar design shows the minimum stress concentration compared to the convergent collar design in both the vertical and oblique loading.

Tables 2-3 show the comparison of von Mises stress (in N/m^2^) in the crestal bone of the CCD and DCD in four densities during vertical and oblique loading (400 N). It can be seen that the divergent collar with D1 density shows the minimum stress while the convergent collar with D3 and D4 densities shows the maximum stress on the vertical load.

**Table 2:** von Mises stress in the crest of the bone during the vertical load in the implant with convergent and divergent collar designs

Bone densities	Divergent	Convergent
D1	3.919e ^+^^06^	5.225e ^+06^
D2	5.064e ^+06^	6.756e ^+06^
D3	6.876e ^+06^	9.161e ^+06^
D4	8.676e ^+06^	1.57e ^+07^

**Table 3:** von Mises stress in the bone crest region during oblique loading in the implant with convergent and divergent collar designs

Bone densities	Divergent	Convergent
D1	7.839e ^+06^	1.045e ^+07^
D2	1.013e ^+07^	1.351e ^+07^
D3	1.375e ^+07^	1.834e ^+07^
D4	1.735e ^+07^	2.314e ^+07^

Overall, the D1 bone with the divergent collar shows the minimum stress concentration and the D4 bone with the convergent collar shows the maximum stress concentration in the crestal bone region during vertical and oblique loading. The stress concentration for vertical loading was lesser than that for oblique loading.

## Discussion

The reason for osseointegration failure and bone loss in the pre-implant is the micromovement of a dental implant that is embedded inside the available bone and the amount of pressure on the bone that is surrounding the endosseous dental implant, as seen by van Steenberghe et al. [15].

The most essential factor to design good quality, successful implants is the density, that is, the quality of the edentulous bone as stated by Holmes and Loftus [16]. In addition to this, successful implant treatment is also based on a patient’s health, implant material and the treatment approach. To reduce the mechanical stress, the DCD and CCD with or without microroughness, microthread and surface treatment were introduced. These collar designs have many variations like straight/parallel side, flared/divergent or tapered/convergent. To cope up with the substantial stress and the bone loss, this research work proposed and designed the most advantageous crest module design. It is a well-known fact that the implants are more successful in the mandible region as it has sufficient volume and density as compared to the maxilla region [13]. Hence, a detailed analysis of the available bone is required before selecting the implant type, surface type and the surgical approach. This pre-operative evaluation can be precisely done by a radiographic examination.

We used a model for finite elemental analysis in a mandibular section of the bone with a dental implant and ceramic crown to observe the effect of the stress on different densities of the available bone by designing a CCD and DCD. Each layer of the finite element model is assigned with some particular materials and characteristics, which can be changed by the stress developed in different bone qualities. FEM is based on some assumptions like loading/boundary conditions and material properties to simulate the architect and surface morphology of the dental implant.

In this study, the use of convergent collar designs showed destructive stresses and pathologic micro-strains around the crestal compact bone under vertical and oblique loading. These collar designs also demonstrated pathologic micro-strains in the crestal compact bone in the low-density cancellous bone under vertical and oblique loading. This can be explained by “saucerization” being particularly prone to occur in the first year of function. The results also showed that there is an increase in the level of stress around the neck of the implant with a decrease in the bone density. This is supported by the study of Sevimay and Turhan [17].

It should be noted that values observed in this FEA study might be worse in actual clinical situations. For a long-term bone maintenance, the divergent implant collar appears to be a favorable choice. By increasing the surface area and decreasing forces, stresses can be reduced as stress is a result of force divided by area. The divergent implant collar had a surface area greater than the convergent collar design and hence it transferred the lowest stress levels to the crestal compact bone under the same load, while the convergent collar had a smaller surface area and transferred the highest stress to the adjacent compact bone [12]. The compact bone is known to be the least resistant to shear forces, which significantly increase by bending overloads from lateral movements. The divergent implant collars transferred lower peak tensile and compressive stresses and strains, while the convergent collars had the opposite effect [12].

FEA is a powerful tool to design an implant to have a better prognosis in prosthodontic treatment planning and rehabilitation [3]. The limitation of the study was the need to simulate the clinical condition of the living bone with various muscular attachments. It was also difficult to match the type of osseointegration occurring clinically around a dental implant at different densities of the bone. At the same time, the masticatory load was not similar to that of the load seen in the living bone, which is yet another limitation. The loads in this study were taken as static while in general, the chewing force is dynamic in nature. The type of osseointegration considered in the model would not match with the cases seen clinically. Therefore, further studies are required to simulate the living bone tissues to evaluate and measure their response to the applied mechanical forces, which could be resolved if advanced imaging techniques are used.

## Conclusions

In this research article, von Mises stresses were seen to be maximum in the bone crest or the neck of the implant for both the convergent and divergent collar implant designs in all the four densities of the bone. The D1 bone model showed the lowest stress concentration compared to D2, D3, and D4. The DCD showed the lower stress and strain concentrations as compared to the CCD in the contiguous crestal bone in all the densities of the bone in both vertical and lateral or oblique loading. The DCD with the D1 bone showed the least stress concentration around the crestal bone region. In the convergent collar implant, the implant crestal module is closer to the bone than in the implants with divergent collars. Therefore, the stress concentration in the convergent collar implant may have a greater effect on crestal bone resorption as compared to that in other collar designs.
